# Polydatin reduces *Staphylococcus aureus* lipoteichoic acid‐induced injury by attenuating reactive oxygen species generation and TLR2‐NFκB signalling

**DOI:** 10.1111/jcmm.13194

**Published:** 2017-05-19

**Authors:** Gan Zhao, Kangfeng Jiang, Haichong Wu, Changwei Qiu, Ganzhen Deng, Xiuli Peng

**Affiliations:** ^1^ Department of Clinical Veterinary Medicine College of Veterinary Medicine Huazhong Agricultural University Wuhan China; ^2^ Key Laboratory of Agricultural Animal Genetics, Breeding and Reproduction Ministry of Education Huazhong Agricultural University Wuhan China

**Keywords:** inflammation, apoptosis, ROS, NF‐κB

## Abstract

*Staphylococcus aureus* (*S. aureus*) causes severe inflammation in various infectious diseases, leading to high mortality. The clinical application of antibiotics has gained a significant curative effect. However, it has led to the emergence of various resistant bacteria. Therefore, in this study, we investigated the protective effect of polydatin (PD), a traditional Chinese medicine extract, on *S. aureus* lipoteichoic acid (LTA)‐induced injury *in vitro* and *in vivo*. First, a significant improvement in the pathological conditions of PD *in vivo* was observed, suggesting that PD had a certain protective effect on LTA‐induced injury in a mouse model. To further explore the underlying mechanisms of this protective effect of PD, LTA‐induced murine macrophages were used in this study. The results have shown that PD could reduce the NF‐κB p65, and IκBα phosphorylation levels increased by LTA, resulting in a decrease in the transcription of pro‐inflammatory factors, such as TNF‐α, IL‐1β and IL‐6. However, LTA can not only activate NF‐κB through the recognition of TLR2 but also increase the level of intracellular reactive oxygen species (ROS), thereby activating NF‐κB signalling. We also detected high levels of ROS that activate caspases 9 and 3 to induce apoptosis. In addition, using a specific NF‐κB inhibitor that could attenuate apoptosis, namely NF‐κB p65, acted as a pro‐apoptotic transcription factor in LTA‐induced murine macrophages. However, PD could inhibit the generation of ROS and NF‐κB p65 activation, suggesting that PD suppressed LTA‐induced injury by attenuating ROS generation and TLR2‐NFκB signalling.

## Introduction

*Staphylococcus aureus* (*S. aureus*) is an opportunistic Gram‐positive bacterium that causes various infectious diseases in humans 1, 2 and animals 3, 4, such as skin and soft‐tissue infections 5, as well as pneumonia 6, sepsis and endometritis 7, and has led to high mortality. LTA is a teichoic acid extracted from the Gram‐positive bacteria cell wall that is the predominant driving force of the host inflammatory response to this type of bacteria 8.

In the physiological state, a balance exists between the production of ROS, including the hydroxyl radical (·OH) and the superoxide radical (O2·^−^) 9, and their neutralization in the system, and no oxidative stress usually occurs 10. Numerous factors, such as LPS and *S. aureus*, induce the significant generation of ROS 11. Oxidative stress condition develops when the balance becomes disturbed and an inequity among pro‐oxidant and antioxidant occurs. The latest studies have shown that oxidative stress plays a significant role in the pathogenesis of many inflammatory diseases 12, 13, and oxidative stress induces apoptosis 14.

Toll‐like receptors (TLRs) are critical for the innate immune system *via* recognizing microbe‐associated molecular patterns (MAMPs) 15, of which LTA from *S. aureus* acting as TLR2‐ligands was recognized by TLR2 16, 17, resulting in the induction of intracellular signalling cascades, including the activation of NF‐κB signalling. However, the transcription factor NF‐κB is crucial in a series of cellular processes, including immune and inflammatory responses and apoptosis 18. Cumulative evidence has indicated that there is an interrelation between ROS and NF‐κB, such that the high intracellular level of ROS could activate NF‐κB. Once activated, NF‐κB can regulate the expression of inflammatory genes and the release of cytokines, including TNF‐α, IL‐1β and IL‐6 19, 20, subsequently inducing apoptosis 21, 22. Apoptosis is a type of cell suicide regulated by a series of complex signalling pathways 23. Cells enter apoptosis upon intracellular damage and certain physiological cues. This is executed by specific cysteine proteases and caspases—for example, the initiator caspases and effector caspases 14.

**Figure 1:**
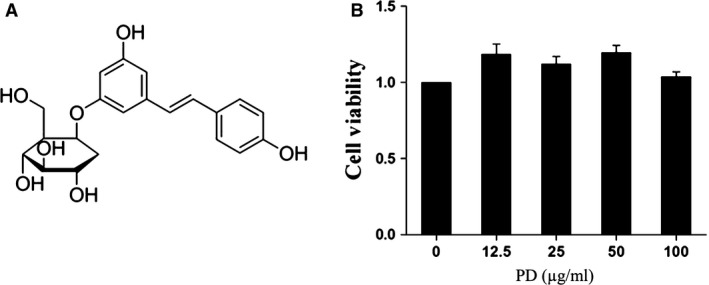
(**A**) Chemical structure of polydatin. (**B**) Effect of polydatin on cell viability. Cells were treated with the indicated concentration of polydatin (0, 12.5, 25, 50, 100 μg/ml) for 24 hrs, and cell viability was detected by CCK‐8 kits.

PD (3,4′‐5‐trihydroxystilbene‐3‐β‐D‐glucopyranoside, shown in Fig. 1A), as a natural precursor of resveratrol, which is a naturally occurring stilbene endowed with multiple health‐promoting effects, is the main active phenolic compound extracted from the root of *Polygonum cuspidatum*, which has been widely used as a traditional Chinese medicine for centuries. Given the potent antioxidant effects 24, anti‐inflammatory effects 25 and antitumour effects 26, it has received worldwide attention for its beneficial effects on cardiovascular, inflammatory, neurodegenerative, metabolic and age‐related diseases 27. Studies have shown that LTA can induce a high level of intracellular ROS in various cell types, leading to injury, such as inflammation 28. However, resveratrol ameliorates inflammation in skeletal muscle cells by attenuating oxidative stress 29, and PD has been shown to ameliorate renal ischaemia/reperfusion injury by decreasing apoptosis and oxidative stress 30. However, it is not known whether PD plays a role in endometritis and its underlying mechanism. Herein, we have been suggested that PD may alleviate LTA from *S. aureus* induced injury by decreasing intracellular ROS levels. Thus, we examined the antagonistic function of PD *in vitro* and *in vivo* and determined the potential therapeutic function of PD in endometritis or other inflammatory diseases.

## Materials and methods

### Chemicals and reagents

PD (purity>99%, Fig. S1) was purchased from the National Institute for the Control of Pharmaceutical and Biological Products (Beijing, China). LTA from *S. aureus* was obtained from Sigma‐Aldrich Chemical Co. (Saint Louis, Missouri, USA). The indicated antibodies, including the NF‐κB Pathway Sampler Kit and Cleaved Caspase Antibody Sampler Kit, were obtained from Cell Signaling Technology (Beverly, MA, USA). 2′,7′‐Dichlorofluorescein diacetate (2′,7′‐DCFH‐DA), One Step TUNEL (terminal deoxynucleotidyl transferase dUTP nick end labelling), Apoptosis Assay Kit and FITC Annexin V Apoptosis Detection Kit with PI (propidium iodide), BAY‐11‐7082 (an inhibitor of NF‐κB) and N‐acetyl‐L‐cysteine (NAC) were obtained from Beyotime Institute of Biotechnology (Shanghai, China). Foetal bovine serum (FBS) was purchased from Sigma‐Aldrich Chemical Co. (Saint Louis, Missouri, USA). All of the other chemicals and reagents were of the highest commercial grade available.

### Animals and cell culture

Six‐ to eight‐week‐old BALB/c mice were obtained from the Animal Experiment Center of Wuhan University (Wuhan, China). All of the experimental procedures involving animals and their care conformed to the Guide for the Care and Use of Laboratory Animals of the National Veterinary Research. This study was approved by the Huazhong Agricultural University Animal Care and Use Committee. The mice were housed in stainless steel cages in an air‐conditioned room in a temperature maintained at 24 ± 1°C and free access to food and water. The collection work was performed under sodium pentobarbital anaesthesia to minimize suffering.

For the *in vivo* assay, the LTA‐induced endometritis mouse model was carried out as follows: six‐ to eight‐week‐old BALB/c mice were randomly divided into five groups (*n* = 6): the control group (CG), LTA group (LTA) and LTA+ PD groups (25, 50 and 100 mg/kg); LTA was dissolved in physiological saline, and the PD stock solution was diluted with physiological saline immediately prior to the experiment. The mice were administered with equal amounts of LTA (5 mg/kg) on each side of the uterus under anaesthesia, and the control group received equal volumes of saline solution. Twenty‐four hours after administration, PD was intraperitoneally injected three times every 8 hrs at dosages of 25, 50 and 100 mg/kg, respectively. The control group and LTA group received equal volumes of intraperitoneal physiological saline. The mice were killed *via* CO_2_ inhalation at 8 hrs after the last injection, and then, the uterine tissues from each group were harvested and immersed in 4% paraformaldehyde; the remaining tissues were stored at −80°C for subsequent experiments.

For the *in vitro* assay, the RAW 264.7 cell lines were obtained from CCTCC (China Center for Type Culture Collection, Wuhan, China). These cells were cultured in RPMI 1640 supplemented with 10% FBS, 2 mM L‐glutamine, 50 U/ml penicillin and 50 μg/ml streptomycin. The cells were maintained in a 5% CO_2_ humidified incubator at 37°C. The cells were treated with LTA alone or in combination with PD or other corresponding treatment. After the treatments, the cells were prepared for further studies.

### Cell viability assay

Cell counting Kit‐8 (CCK‐8, Dojindo Laboratories, Minato‐ku, Tokyo, Japan) was used to assess cell viability. The RAW 264.7 cells were seeded in 96‐well cell culture plates at a density of 2 × 10^4^ cells/ml. After culture with different concentrations of PD (12.5, 25, 50 or 100 μg/ml) for 24 hrs, cells were continuously cultured with 10 μl of CCK8 in each well at 37°C for 2 hrs. Cell viabilities were measured through absorbance (optical density) with a microplate reader (Bio‐Rad Instruments, Hercules, CA, USA) at 450 nm. Cell viability = (Treatment Group OD‐Blank Group OD)/(Control Group OD‐Blank Group OD).

### Histological assay

The uterine tissues from each group were harvested and immersed in 4% paraformaldehyde, embedded in paraffin, cut into 4‐μm sections, stained with haematoxylin/eosin (H&E) and then were examined under a microscope (Olympus Shinjuku‐ku, Tokyo, Japan).

### RNA extraction and qPCR

Total RNA was isolated by TRIzol (Invitrogen, Carlsbad, California, USA). The total RNA was treated with DNase I and reverse‐transcribed using oligo‐dT primers. The total cDNA was used as the starting material for real‐time PCR with FastStart Universal SYBR Green Master (Roche Applied Science, Mannheim, Germany) Germany) using the StepOne real‐time PCR System (Life Technologies Corp. Waltham, MA USA). The Primer Premier software (PREMIER Biosoft International, Palo Alto, California, USA) was used to design specific primers for TNF‐α, IL‐1β and IL‐6 and GAPDH based on known sequences (Table 1). The expression levels of each target gene were normalized to the corresponding GAPDH threshold cycle (CT) values using the 2^−▵▵CT^ comparative method.

**Table 1:** Oligonucleotide primers used for qPCR

Name	Accession number	Primer sequence (5′–3′)	Product size(bp)
TLR2	NM_011905.3	Forward:TCTAAAGTCGATCCGCGACAT Reverse:CTACGGGCAGTGGTGAAAACT	155
TNF‐α	NM_013693.3	Forward:CTTCTCATTCCTGCTTGTG Reverse:ACTTGGTGGTTTGCTACG	198
IL‐1β	NM_008361.4	Forward:CCTGGGCTGTCCTGATGAGAG Reverse:TCCACGGGAAAGACACAGGTA	131
IL‐6	NM_031168.1	Forward:GGCGGATCGGATGTTGTGAT Reverse:GGACCCCAGACAATCGGTTG	199
GAPDH	NM_001289726.1	Forward:CAATGTGTCCGTCGTGGATCT Reverse:GTCCTCAGTGTAGCCCAAGATG	124

### siRNA transfection

The siRNA of TLR2 (si‐TLR2) and its negative control (si‐NC) were designed and synthesized (RiboBio Co., Guangzhou, China). The synthetics were transfected into RAW 264.7 cells at the final concentration of 200 nM using Lipofectamine 2000 (Invitrogen, Carlsbad, California,USA) according to the manufacturer's instructions. The whole transfection process was proceeded in a non‐serum medium named opti‐MEM (Gibco, Gaithersburg, MD, USA) for 6 hrs at 37°C in a humidified environment containing 5% CO_2_. After transfection, the medium was changed into a previous medium. For the LTA group, cells were treated with LTA (5 μg/ml) for 3 hrs, and the PD treatment groups were pre‐treated with PD at the dose of 50 μg/ml for 1 hr, and then, LTA (5 μg/ml) was added for 3 hrs. For the H_2_O_2_ (400 μM) 24 group, similar processing was performed with cells lysis for further study.

### Western blot analysis

Total protein of the tissues and cells was extracted according to the manufacturer's recommended protocol (Vazyme, Nanjing, China). The protein concentrations were determined using the BCA Protein Assay Kit (Vazyme, Nanjing, China). Samples with equal amounts of protein (50 μg) were fractionated on 10% SDS–polyacrylamide gels, transferred to polyvinylidene difluoride membranes and blocked in 5% skim milk in TBST for 1.5 hrs at 25 ± 1°C. The membranes were then incubated at 4°C overnight with 1:1000 dilutions (v/v) of the primary antibodies. After washing the membranes with TBST, incubations with 1:4000 dilutions (v/v) of the secondary antibodies were conducted for 2 hrs at 25 ± 1°C. Protein expression was detected using an Enhanced Chemiluminescence Detection System. β‐Actin was used as a loading control.

### NF‐κB p65 immunofluorescence assay

Tissues were analysed on 4‐μm paraffin sections using antigen retrieval for 10 min. or 5 min. of boiling in 10 mM citrate buffer, pH 6.0. Cultured cells were fixed in 4% paraformaldehyde (pH 7.4) or methanol at −20°C for 3 min. and then washed four times in PBS. Cells or sections were permeabilized with 0.1% Triton X‐100, exposed to the blocking solution (PBS/3% BSA) and incubated with the primary antibodies NF‐κB p65 at 4°C overnight. After four washes in PBS, the cells were incubated with secondary fluorescently labelled antibodies Dylight 594 antibodies for 45 min. at RT and then were washed three times in PBS. Nuclei were stained using DAPI. Fluorescent images were taken using an AX70 widefield microscope (Olympus). All morphometric measurements were observed by at least three independent individuals in a blinded manner.

### Measurement of ROS production

ROS levels were determined by measuring the oxidative conversion of cell permeable 2′,7′‐dichlorofluorescein diacetate (DCFH‐DA) to fluorescent dichlorofluorescein (DCF). Cells in six‐well culture dishes were incubated with control media or 10 μg/ml LTA for 3 hrs in the absence or presence of PD (12.5, 25, 50 μg/ml) or NAC (500 μM). The cells were washed with D‐Hank's and incubated with DCFH‐DA at 37°C for 30 min. Next, DCF fluorescence was observed under the microscope (Leica, Wetzlar, Germany), and intracellular ROS fluorescence intensity was assessed by IOD (Integrated option density)/area through Image‐Pro Plus 6.0 image analysis software (Media Cybernetics, Washington, MD, USA).

### TUNEL assay

Tissues were performed on 4‐μm paraffin sections using antigen retrieval for 10 min. or 5 min. of boiling in 10 mM citrate buffer (pH 6.0). Cells in 6‐well culture dishes were incubated with control media or 10 μg/ml LTA for 3 hrs in the absence or presence of PD (12.5, 25, 50 μg/ml) or NAC (500 μM). They were next fixed in 4% paraformaldehyde (pH 7.4) or methanol at −20°C for 3 min. and then washed four times in PBS. Cells or sections were permeabilized with 0.1% Triton X‐100. After washing with PBS, samples were first incubated with a terminal deoxynucleotide transferase‐mediated dUTP nick end labelling (TUNEL) reagent containing terminal deoxynucleotidyl transferase and fluorescent isothiocyanate‐dUTP. They were then stained with 1 μg/ml DAPI for 30 min. to evaluate the cell nucleus by UV light microscopic observations (blue). Samples were analysed in a drop of PBS under a fluorescence and UV light microscope. All morphometric measurements were observed by at least three independent individuals in a blinded manner.

### Flow cytometry

To further corroborate the effect of PD on apoptosis induced by LTA, Annexin V and PI double staining was detected by flow cytometry. Briefly, cells (5 × 10^5^ cells/well) cultured in six‐well plates were incubated with control media or 10 μg/ml LTA for 3 hrs in the absence or presence of PD (12.5, 25, 50 μg/ml). At the end of treatment, the cells were harvested, washed twice with cold PBS, adjusted to 100 μL of 1 × Annexin V binding buffer (1 × 10^5^ cells) and transferred to a 5‐ml culture tube. Next, 5 μL of Annexin V‐FITC and 5 μL of PI was added, and the cells were gently vortexed. The cells were then incubated in the dark for 15 min. at room temperature (25°C). The apoptosis rates were determined using a FACSCalibur flow cytometer (Becton Dickinson, Franklin Lakes, New Jersey, USA) after the addition of 400 μL of 1 × binding buffer.

### Statistical analysis

All experiments were three independent repeats, and the results were analysed using GraphPad Prism 5 (GraphPad InStat Software, La Jolla, CA , USA). Comparisons among all groups were performed with one‐way anova. The data were expressed as means  ±  S.E.M. *P* values <0.05 were considered to be statistically significantly different.

## Results

### Effect of PD on cell viability

To investigate whether the current PD experimental concentration has an effect on the viability of cells, cell viability assays were conducted using the CCK‐8 kit. The data showed there was little effect on the cell viability of RAW 264.7 cells treated with the indicated concentration of PD (shown in Fig. 1B).

### Effect of PD on LTA‐induced injury in a mouse model

**Figure 2:**
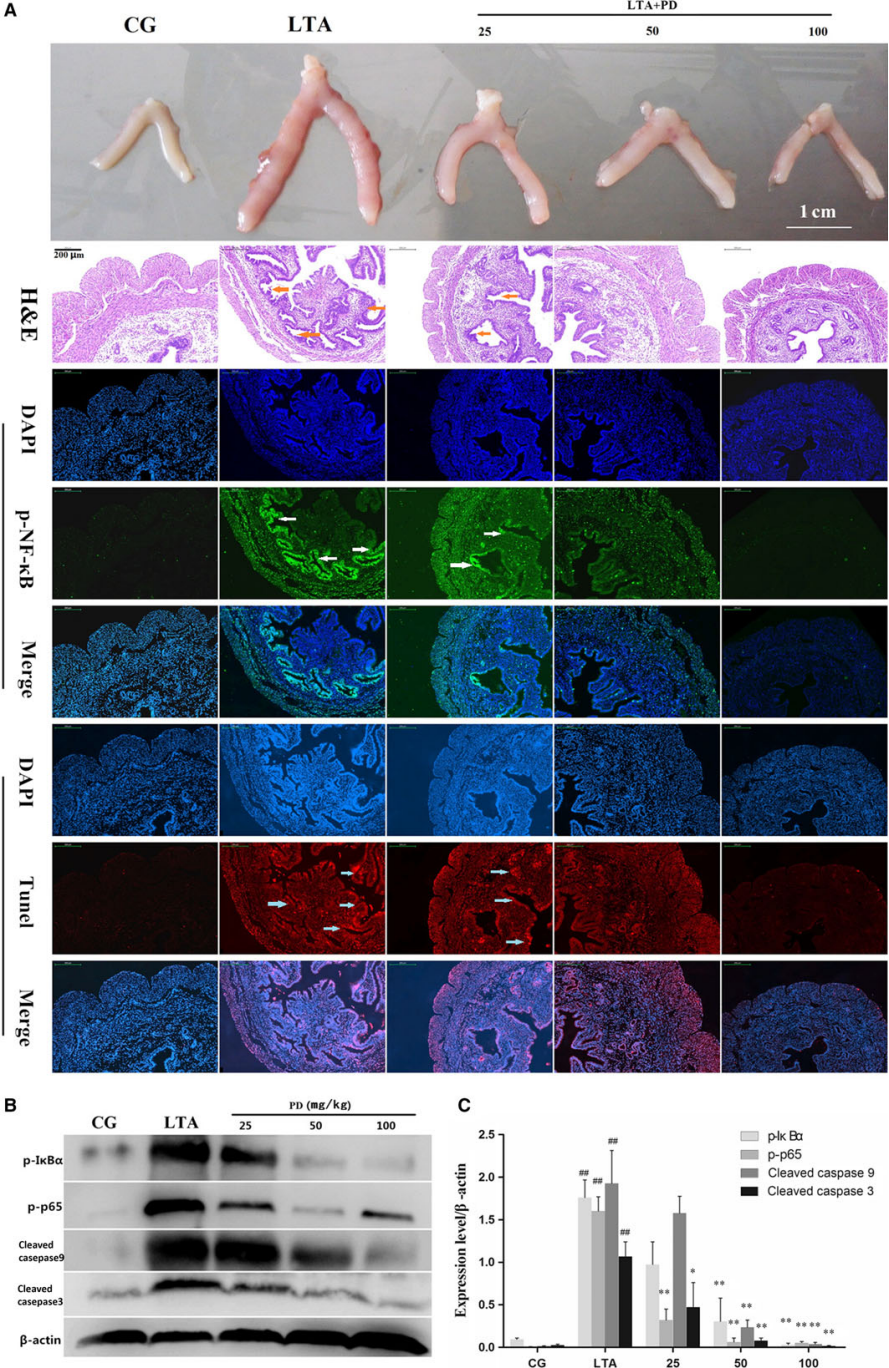
(**A**) Histological examination of the protective effect of polydatin on LTA‐induced uterine injury in mice (*n* = 6). From top to bottom: uterine morphology observation, scale bar: 1 cm; H&E staining of uterine tissue; phosphorylated NF‐κB p65 immunofluorescence staining (Green) of uterine tissue; TUNEL staining of uterine tissue. Cell nuclei (Blue), TUNEL‐positive cells (Red). Scale bar: 200 μm. The red, white and blue arrows indicate the tissue lesion, the translocation of p65 and the apoptotic region, respectively. (**B**) The protein levels of phosphorylated NF‐κB p65 (p‐p65), phosphorylated IκBα (p‐IκBα) and cleaved caspases 9 and 3 were determined by Western blotting. β‐Actin was used as an internal control. (**C**) The Western blotting data were represented the means ± S.E.M. of three independent experiments. CG is the control group, LTA is the LTA group, and 25, 50 and 100 are the polydatin‐treatment groups representing 25 mg/kg, 50 mg/kg and 100 mg/kg per animal, respectively. ^#^ *P* < 0.05, ^##^ *P* < 0.01 versus the CG group. *P < 0.05, **P < 0.01 versus the LTA group.

In this study, four mice in each group (*n* = 6) were randomly selected for analysis of the following analysis, including H&E, NF‐κB p65 immunofluorescence, TUNEL staining, and Western blot. We found that administration with LTA resulted in severe inflammation, manifesting as inflammatory cell infiltration, increased uterine cavity effusion and uterine epithelial cell detachment, and necrosis. However, treatment with PD (50, 100 mg/kg) evidently reduced the pathological conditions (shown in Fig. 2A). Nuclear transcription factor κB (NF‐κB) is involved in the transcription and modulation of several inflammatory mediator genes and plays an important role in the inflammatory process. Thus, the phosphorylation level of NF‐κB p65 was detected by immunofluorescence assay, and further confirmation was conducted by Western blotting. The results showed a marked increase in the phosphorylation of IκBα and NF‐κB p65 induced by LTA, which was inhibited by PD treatment in a dose‐dependent manner (as shown in Fig. 2B, 2C). NF‐κB, however, is crucial in a series of cellular processes, including immune and inflammatory responses, and apoptosis 18. To investigate the effect of PD on LTA‐induced apoptosis in mice, the TUNEL assay and caspase 3, 9 activities were assessed in this research. Interestingly, PD effectively reduced the apoptosis induced by LTA treatment (as shown in Fig. 2). These results indicated that PD effectively reduced LTA‐induced injury *in vivo*, such as the protective of apoptosis and inflammation.

### PD reduces LTA‐induced apoptosis in RAW 264.7 cells

**Figure 3:**
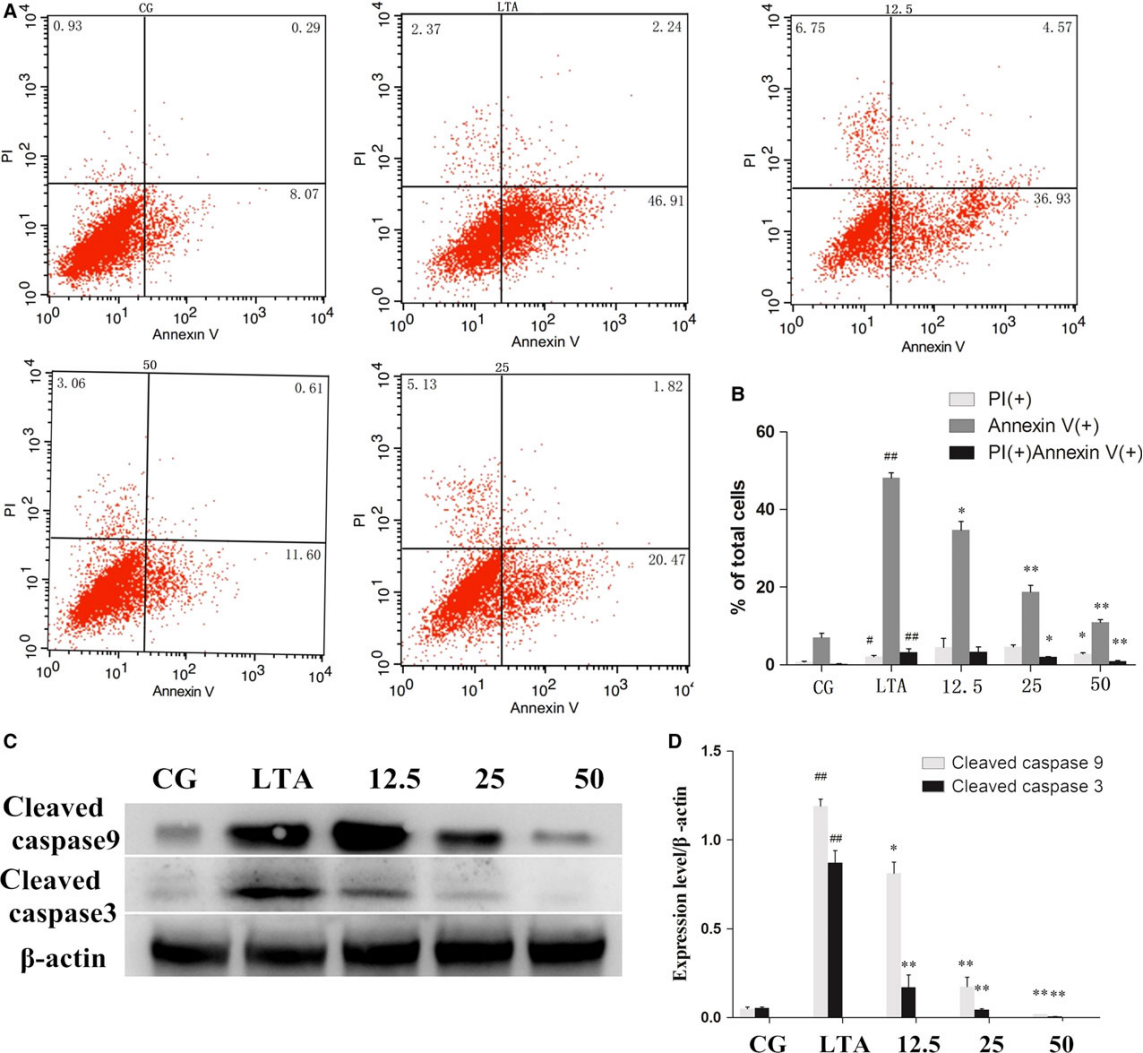
Effect of polydatin on apoptosis induced by LTA. (**A**) Representative dot plots of staining with Annexin V and PI. Cells were treated as described previously. (**B**) Numbers in the quadrants are the percentages of each population. The data are represented as the means ± S.E.M. of three independent experiments. (**C**) The protein levels of cleaved caspases 9 and 3 were determined by Western blotting. β‐Actin was used as an internal control. (**D**) The Western blotting data were represented as the means ± S.E.M. of three independent experiments. CG is the control group, LTA is the LTA group, and 12.5, 25 and 50 are the polydatin‐treatment groups representing 12.5 μg/ml, 25 μg/ml and 50 μg/ml per cell plate, respectively. ^#^ *P* < 0.05, ^##^ *P* < 0.01 versus the CG group. *P < 0.05, **P < 0.01 versus the LTA group.

*In vivo* experiments revealed that PD may have a potential anti‐inflammatory and anti‐apoptotic effect. To further confirm these phenomena, *in vitro* experiments were carried out. We examined whether PD exhibited an anti‐apoptotic effect in RAW 264.7 cells exposed to high concentrations of LTA (10 μg/ml). Flow cytometry analysis showed that LTA profoundly triggered apoptosis (Fig. 3A), while PD remarkably decreased the percentage of apoptotic cells (Fig. 3A, B). The inhibitory effect of PD on apoptosis was further confirmed by a reduction in caspase‐3 and caspase‐9 activation in LTA‐stimulated RAW264.7 cells. The results showed that LTA stimulated the activation of caspases 3 and 9 and that the LTA triggered the activation of executioner caspases in a dose‐dependent manner (Fig. 3C, D). These results indicated that PD also plays an anti‐apoptotic role in LTA‐stimulated RAW 264.7 cells.

### PD attenuates LTA‐induced ROS production

**Figure 4:**
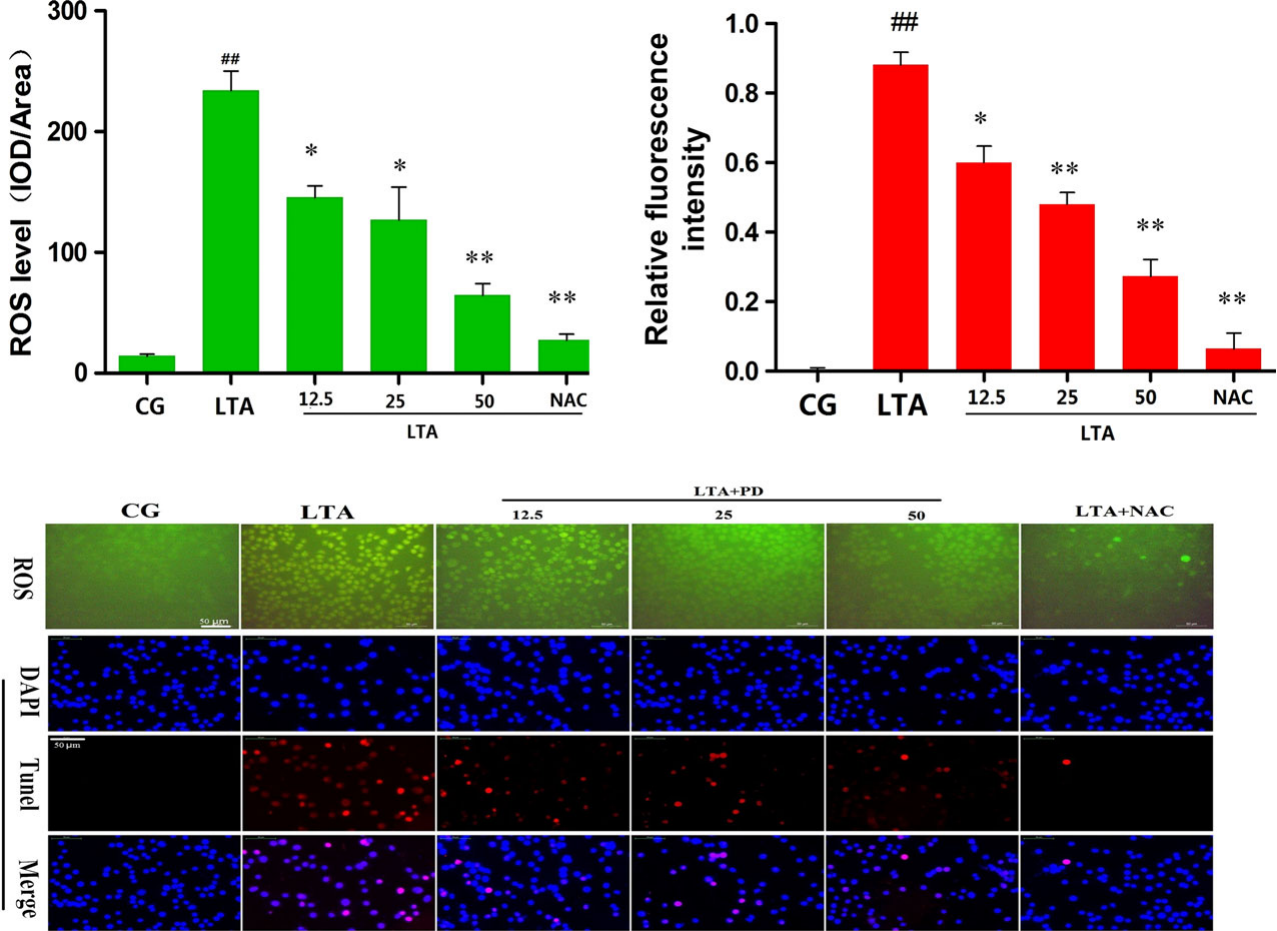
Fluorescence microscopy of ROS production by DCFH‐DA (green) after stimulation or treatment (ROS). Inhibition of LTA‐induced cell apoptosis by polydatin was examined by the TUNEL assay (TUNEL). Blue spots represent cell nuclei, and red spots represent TUNEL‐positive cells. CG is the control group, LTA is the LTA group, and 12.5, 25 and 50 are the polydatin‐treatment groups representing 12.5 μg/ml, 25 μg/ml and 50 μg/ml per cell plate, respectively. NAC is the NAC (500 μM)‐treatment group. The integrated option density (IOD) of DAPI was used as an internal control. All of the data represent the mean ± S.E.M. of three independent experiments. ^#^ *P* < 0.05, ^##^ *P* < 0.01 versus the CG group. *P < 0.05, **P < 0.01 versus the positive LTA group.

Studies have revealed that oxidative stress could cause cellular apoptosis *via* various pathways, including mitochondria‐dependent and mitochondria‐independent pathways 14. Thus, we determined the ROS level in LTA‐induced RAW 264.7 cells. As shown in Figure 4, the level of ROS was significantly increased with the LTA treatment, which was attenuated by PD in a dose‐dependent manner. Additionally, this increase was abolished by the antioxidant NAC (500 μM). To investigate whether ROS induces apoptosis, the apoptosis condition was detected by TUNEL assay, and the results showed the apoptosis condition was in accordance with the intracellular ROS level, and the NAC also attenuated the LTA‐induced apoptosis level (shown in Fig. 4), indicating that PD may reduce apoptosis in RAW 264.7 cells *via* attenuating LTA‐induced ROS production.

### PD reduces the TLR2‐dependent or TLR2‐independent NF‐κB signalling pathway

**Figure 5:**
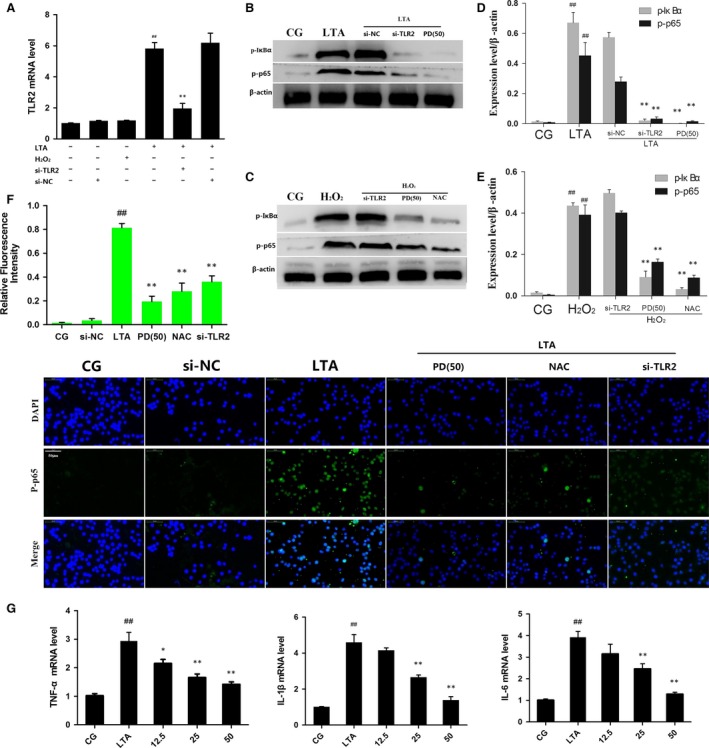
(**A**) The interfering efficiency of TLR2 siRNA and effect of LTA or H_2_O_2_ on TLR2 expression were measured by RT‐PCR. (**B**) The protein levels of p‐p65 and p‐IκBα were stimulated with LTA after the knockdown of TLR2 by siRNA or PD pre‐treatment. (**C**) The protein levels of p‐p65 and p‐IκBα were stimulated with H_2_O_2_ after the knockdown of TLR2 by siRNA or PD or NAC pre‐treatment. β‐Actin was used as an internal control. (**D**,** E**) The Western blotting data were represented as the means ± S.E.M. of three independent experiments. (**F**) Translocation of the p65 subunit from the cytoplasm into the nucleus was evaluated by immunofluorescence. Blue spots represent cell nuclei, and green spots represent p‐p65 staining. The integrated option density (IOD) of DAPI was used as an internal control. (**G**) The effect of polydatin on the mRNA levels of IL‐1β, IL‐6 and TNF‐α induced by LTA was determined by qPCR in RAW 264.7 cells. *GAPDH* was used as a control. All of the data were represented as the means ± S.E.M. of three independent experiments. CG: Control group, LTA: LTA group, NAC: NAC‐treatment group, H_2_O_2:_ H_2_O_2_ group, si‐TLR2: TLR2 siRNA, si‐NC: TLR2 siRNA negative control; PD(50): cells treated with polydatin with a concentration of 25 μg/ml, and 12.5, 25 and 50 represent the polydatin‐treatment groups representing 12.5 μg/ml, 25 μg/ml and 50 μg/ml per cell plate, respectively. Data represent the mean ± S.E.M. of three independent experiments. ^#^ *P* < 0.05, ^##^ *P* < 0.01 versus the CG group. *P < 0.05, **P < 0.01 versus the positive group (LTA or H_2_O_2_).

Research has been revealed that LTA from *S. aureus* acting as a TLR2‐ligand was recognized by TLR2 16, 17, leading to the activation of transcription factors, such as NF‐κB, which was required for the expression of inflammatory cytokines 31. Thus, to investigate whether the activation of NF‐κB is TLR2‐dependent, specific TLR2‐blocked siRNA (si‐TLR2) was used, and then, the phosphorylation of NF‐κB p65 and IκBα in RAW 264.7 cells that had been exposed to LTA or H_2_O_2_ was examined. The results showed that LTA induced high expression of TLR2 that was decreased by si‐TLR2; however, H_2_O_2_ treatment did not affect the expression of TLR2 (Fig. 5A). Interestingly, similar results were observed in the determination of the phosphorylation of NF‐κB p65 and IκBα—that is, the phosphorylation of NF‐κB p65 and IκBα induced by LTA was attenuated by si‐TLR2 and PD (50 μg/ml) (Fig. 5B). Recently, studies have shown the high intracellular level of ROS could activate NF‐κB and subsequently regulate the downstream biological processes 32. Thus, some clinical drugs were developed to mitigate inflammation by abrogating the state of oxidative stress 33. H_2_O_2_, as a type of strong oxidant, can significantly increase the intracellular ROS level^25^. Interestingly, our results revealed that H_2_O_2_ also induced an increase in NF‐κB p65 and IκBα, which was abolished by NAC; however, pre‐treatment with si‐TLR2 alone did not affect these phosphorylation levels, and LTA induced NF‐κB signalling activation *via* TLR2 or inhibition *via* PD treatment (50 μg/ml) (Fig. 5C).

To further confirm the effect of PD on the activation of NF‐κB, the nuclear translocation of NF‐κB was detected by immunofluorescence assay. As shown in Fig. 5F, immunostaining for the phosphorylated NF‐κB p65 (p‐p65) revealed that 3 hrs of exposure to LTA (5 μg/ml) induced the translocation of NF‐κB from the cytosol to the nucleus. However, PD (50 μg/ml) treatment as well as exposure to NAC (500 μM) and si‐TLR2 effectively blocked the nuclear translocation of NF‐κB. In addition, the LTA‐induced increase in the mRNA levels of NF‐κB downstream cytokines (IL‐6, TNF‐α, IL‐1β) was attenuated by PD in a dose‐dependent manner (Fig. 5G), suggesting that PD reduced the NF‐κB signalling pathway in a TLR2‐dependent or TLR2‐independent manner.

### NF‐κB is involved in LTA‐induced apoptosis in RAW 264.7 cells

**Figure 6:**
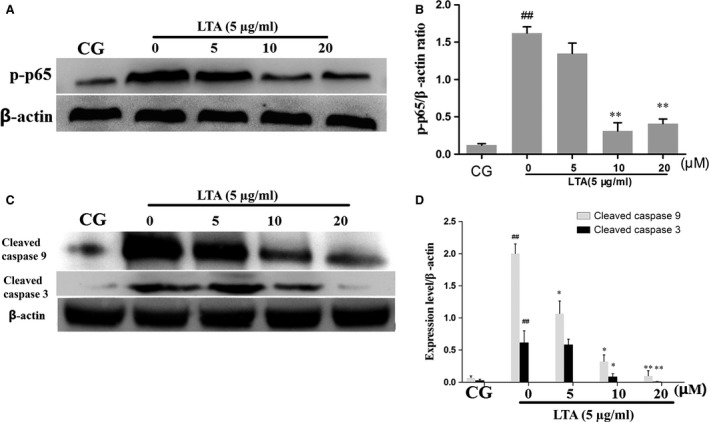
(**A**) The protein levels of p‐p65 stimulated with LTA after blockade by BAY‐11‐7082 with the indicated concentration. β‐Actin was used as an internal control. (**C**) The protein levels of cleaved caspases 9 and 3 stimulated with LTA after blockade by BAY‐11‐7082 with the indicated concentrations. β‐Actin was used as an internal control. (**B**,** D**) The Western blotting data were represented as the means ± S.E.M. of three independent experiments. CG is the control group, LTA is the LTA group, and 0, 5, 10, 20 are the BAY‐11‐7082‐treatment groups representing the concentrations of 0, 5, 10 and 20 μM per cell plate, respectively. ^#^ *P* < 0.05, ^##^ *P* < 0.01 versus the CG group. *P < 0.05, **P < 0.01 versus the LTA group.

NF‐κB was demonstrated to act as a critical regulator involved in apoptosis in various cell types 34, 35. TLR2 blockade led to a decreased level of apoptosis induced by LTA that was also attenuated by NF‐κB signalling, suggesting that NF‐κB was involved in LTA‐induced apoptosis in RAW 264.7 cells. Next, we blocked NF‐κB using a specific NF‐κB inhibitor. Briefly, cells were pre‐treated with BAY‐11‐7082 (5, 10, 20 μM) for 1 hr and then exposed to LTA (5 μg/ml). First, due to NF‐κB inhibitor treatment, the results showed decreased phosphorylation levels of NF‐κB p65 in a dose‐dependent manner, and then, apoptosis was detected by caspase 3, 9 activity. The results showed that LTA activated caspase 3, 9, which was effectively attenuated by BAY‐11‐7082 cotreatment in a dose‐dependent manner (Fig. 6). These results indicated that LTA induced NF‐κB activation, which acts as a critical regulator involved in apoptosis.

## Discussion

*Staphylococcus aureus*, a typical representative of Gram‐positive bacteria, is one of the major pathogens of many human and animal inflammatory diseases, including endometritis 7, 36. LTA, a specific endotoxin embedded in the cell wall of *S. aureus*, has been reported to activate the inflammatory response 37. Although PD has been showed a potent anti‐inflammatory effects 25, previous studies have focused on the inhibition of pro‐inflammatory factors to exert anti‐inflammatory effects, Recently, it has been found that the level of intracellular ROS also causes the tissue injury in many inflammatory disease, such as atherosclerosis 38, 39. However, our study has confirmed PD may play a protective role by reducing intracellular ROS levels, which might provide a new therapeutic target for the development of anti‐inflammatory drugs. In this study, LTA purified from *S*. *aureus* was used to mimic the inflammation, and a mouse model of LTA‐induced endometritis was successfully established. Next, we evaluated the potential protective effects of PD on LTA induced injury. The data showed that the anti‐inflammatory and anti‐apoptotic effects of PD *in vivo* were observed, in agreement with the results of a previous study 40. Next, macrophages were used to explore the deep‐seated mechanism of PD *in vitro*.

Macrophages as important immune cells involved in the regulation of numerous chronic inflammatory diseases, infectious disorders by the secretion of a series of pro‐inflammatory cytokines and chemokines 41, 42. And are widely used as an inflammation model to evaluate the potential protection of a drug *in vitro* 43, 44. The excessive production of pro‐inflammatory cytokines increases the immune response, which in turn results in inflammatory cascade and tissue injury 45, 46.Thus, inhibiting the release of inflammatory cytokines may be a target for anti‐inflammatory drug therapies. Therefore, in this study, we used macrophages instead of primary endometrial epithelial cells to explore the underlying mechanism of PD, which could have more general applicability—that is, PD may also play a similar role in other inflammatory diseases that have been confirmed in our previous studies 47. In this research, we evaluated the protective effects of PD *in vivo* using histological analyses, including H&E, immunofluorescence staining of phosphorylated NF‐κB involved in the regulation of the inflammatory process 48 and the TUNEL assay as well as some of the crucial apoptosis‐related proteins. All of the *in vivo* experiments showed that PD can ameliorate the pathological conditions and attenuate the phosphorylation of NF‐κB and anti‐apoptotic effect, indicating that PD may have potential anti‐inflammatory and anti‐apoptotic effects in LTA‐induced injury *in vivo*.

Although it was previously reported that PD exerted anti‐inflammatory effects *via* inhibiting the phosphorylation of NF‐κB 49, 50, the activation of NF‐κB was induced by various factors, including lipopolysaccharide (LPS) and LTA, which act as TLR ligands, subsequently activating NF‐κB mediated by TLRs 51. However, using siRNA that specifically blocks TLR2 showed that the activation of NF‐κB is not only dependent on TLR2. LTA can induce high levels of intracellular ROS, leading to the activation of NF‐κB, which may be due to the high levels of intracellular ROS and to the phosphorylation of IκBα, a target gene of NF‐κB and the subsequent degradation of IκBα, resulting in the activation of the NF‐κB pathway 52, 53. Our findings suggest that NF‐κB activation involves a new mechanism that is completely different from those triggered by pro‐inflammatory cytokines.

The transcription factor NF‐κB participates in many biological processes such as immunity, inflammation and apoptosis 54. Under normal physiological conditions, NF‐κB is sequestered in the cytoplasm as an inactive form complexed with an inhibitory IκBα protein. Once stimulated with various TLR ligands, IκBα is phosphorylated. The phosphorylation targets IκBα for ubiquitination and degradation, resulting in the translocation of NF‐κB from the cytoplasm into the nucleus and its binding to the κB site in target promoters 51, followed by the regulation of downstream gene expression, including those encoding pro‐inflammatory cytokines, and antioxidant‐ 55 and apoptosis‐related proteins 56. We showed similar results in the present study. LTA induced the activation of NF‐κB, as detected by Western blotting and immunofluorescence staining of phosphorylated NF‐κB p65 in a TLR2‐dependent or TLR2‐independent manner that was attenuated by PD as well as downstream pro‐inflammatory gene expression. To determine whether NF‐κB is also involved in LTA‐induced apoptosis, an inhibitor of NF‐κB (BAY‐11‐7082) 57, 58 was used in this study, and TUNEL assay confirmed the hypothesis. However, NF‐κB is a double‐edged sword; it is involved in the regulation of both pro‐ and anti‐apoptosis. Specifically, some inducers of NF‐κB result in the repression of anti‐apoptotic genes and the induction of pro‐apoptotic genes 56; which of these regulation processes dominates will probably depend on the cell type and nature of the inducing stimulus.

**Figure 7:**
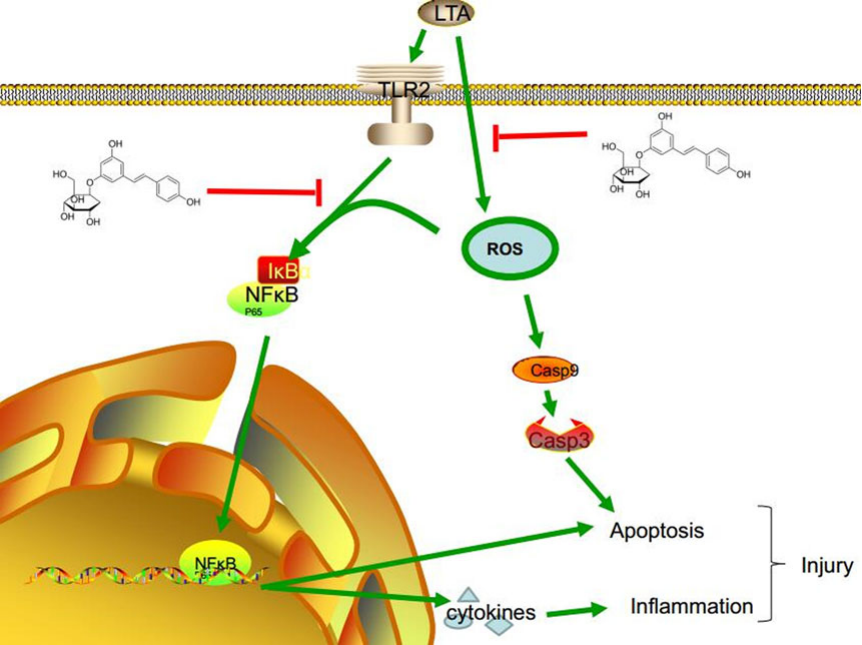
Schematic diagram of a signalling pathway related to anti‐apoptotic or anti‐inflammatory effects of polydatin on LTA‐induced injury. LTA can induce NFκB activation in a TLR2‐dependent or TLR2‐independent manner, leading to the release of downstream pro‐inflammatory cytokines. Moreover, LTA can increase the level of intracellular ROS, which induce apoptosis *via* activating caspases 9 and 3. In addition, NFκB acts as a pro‐apoptotic regulator involved in apoptosis signalling. However, the treatment of PD can suppress LTA‐induced injury by attenuating ROS generation and TLR2‐NFκB activation.

Accumulated evidence has indicated that ROS plays crucial roles in the determination of cell fate as second messengers, and by modifying various signalling molecules 59, apoptosis signal pathways are involved 60. Apoptosis is a regulated physiological process leading to cell death. Caspases, a family of cysteine acid proteases, including initiator caspases and effector caspases, are central regulators of apoptosis. Caspase 9 and caspase 3, which act as a crucial initiator caspase and effector caspase, respectively, have been reported to be activated by ROS 61. Caspase 9 is closely coupled to proapoptotic signals. Once activated, it cleaves and activates downstream effector caspases, such as caspase 3 to induce apoptosis 14, 62. Our results showed that PD treatment could inhibit LTA‐induced apoptosis through the activation of caspases 9 and 3. A previous study has reported that PD attenuates H_2_O_2_‐induced oxidative stress 63. Thus, we also confirmed whether PD inhibits LTA‐induced apoptosis through the ROS‐dependent activation of caspases. NAC, as a common antioxidant, was used to block ROS generation 64, and H_2_O_2_‐induced oxidative stress was used as a positive control 63. The results demonstrated that after treatment with NAC or PD (50 μg/ml), the intracellular ROS level and the caspase survival signals were attenuated significantly (9,3), which have also been shown to be mediated *via* the activation of the NF‐κB pathway 54. Interestingly, after blocking the expression of TLR2, the level of ROS and the apoptosis conditions were also attenuated, which may be due to the restriction of the TLR2‐dependent activation of NF‐κB. Therefore, PD can inhibit apoptosis *via* attenuating ROS‐dependent activation of caspases 9 and 3 (Fig. 7).

In summary, we show here that PD can exert potential protective effects on LTA‐induced injury in both *in vitro* and *in vivo* systems and may occur *via* the attenuation of ROS generation and TLR2‐NFκB signalling. Therefore, PD can possess the potential to be developed as a therapeutic medicine to prevent inflammation diseases, such as *S. aureus* infections, or other oxidative stress damage.

## Conflict of Interest

The authors declare no conflict of interest.

## Author's Contribution

G.Z., X.P. and G.D. conceived and designed the experiments. G.Z., K.J., H.W. and C.Q. performed the experiments. G.Z., K.J., H.W. and G.D. analysed the data. G.Z., K.J. and G.D. wrote the manuscript. All of the authors read and approved the final manuscript.

## Supporting information


**Fig. S1** The purity of PD was determined by high performance liquid chromatography (HPLC).
