# Supplementary material for: Polydatin reduces Staphylococcus aureus lipoteichoic acid‐induced injury by attenuating reactive oxygen species generation and TLR2‐NFκB signalling
Source: J Cell Mol Med. 2017 May 19;21(11):2796–808. doi: 10.1111/jcmm.13194 (PMC5661256; doi:10.1111/jcmm.13194)
Supplement: Supplementary file 1 — Fig. S1 The purity of PD was determined by high performance liquid chromatography (HPLC). [file JCMM-21-2796-s001.docx]

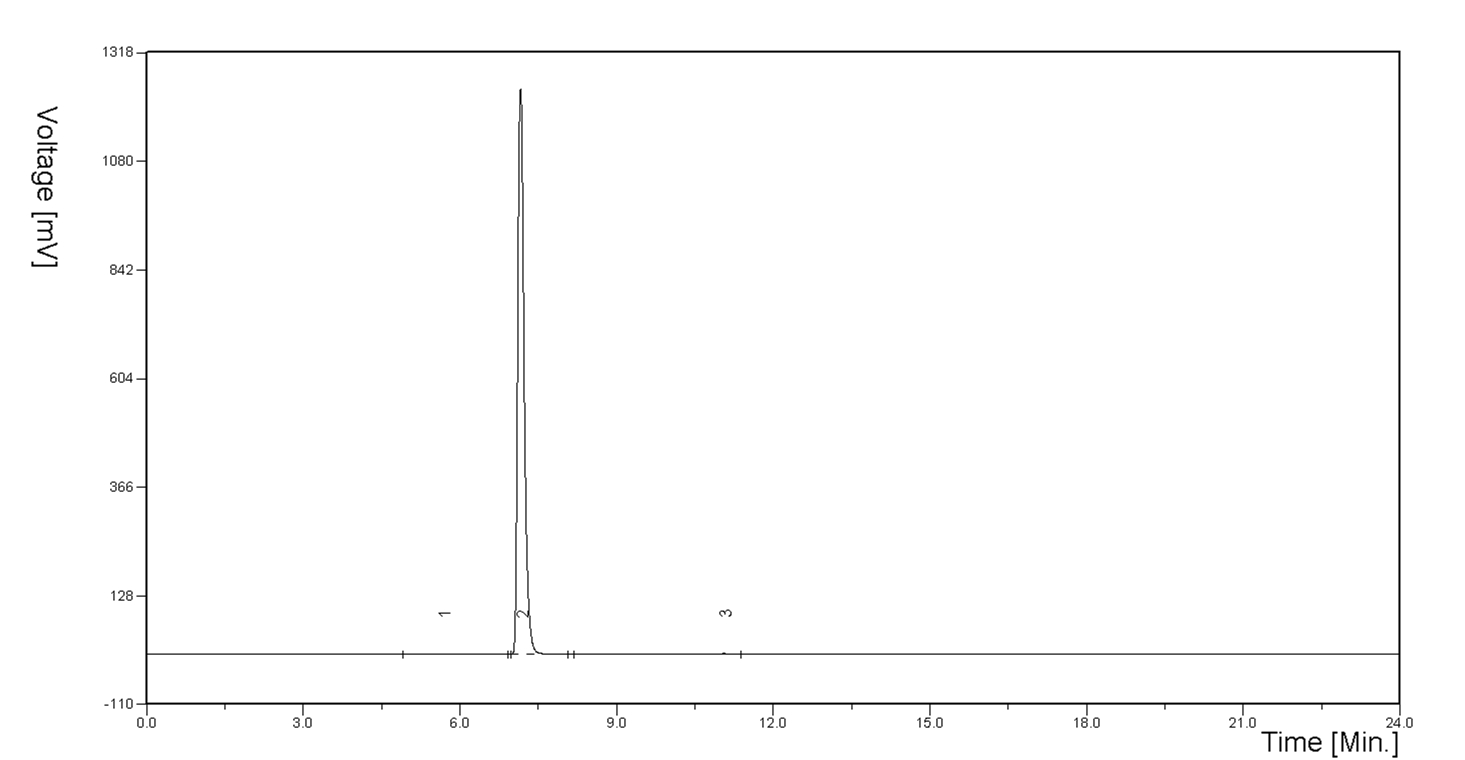


**Fig.1** The purity of PD was determined by high performance liquid chromatography (HPLC).

The experiment was performed on EChrom2000 DAD Data System (Elite, Dalian, China). Chromatography was performed through a Hyper ODS2 C18 column (5 μm, 250×4.6 mm, Dikma technology, USA ). Elution was carried out with acetonitrile/water (74:26), and the flow rate was 1.0 mL/min with DAD detection at 315 nm.  There were 3 peaks were observed in Fig 1, of which, peak 2 (Time = 7.16 min, Voltage = 1238.46 mV) represented the PD with a peak area ratio of 99.71%. which means that the purity of PD can reach more than 99%.
